# Pre-treatment neutrophil to lymphocyte ratio may be a useful tool in predicting survival in early triple negative breast cancer patients

**DOI:** 10.1186/s12885-015-1204-2

**Published:** 2015-03-28

**Authors:** Mirco Pistelli, Mariagrazia De Lisa, Zelmira Ballatore, Miriam Caramanti, Alessandra Pagliacci, Nicola Battelli, Francesca Ridolfi, Matteo Santoni, Elena Maccaroni, Raffaella Bracci, Alfredo Santinelli, Tommasina Biscotti, Rossana Berardi, Stefano Cascinu

**Affiliations:** 1 Clinica di Oncologia Medica, Università Politecnica delle Marche, Ancona, AO Ospedali Riuniti-Ancona, Italy; 2 Anatomia Patologica, AO Ospedali Riuniti-Ancona, Università Politecnica delle Marche, Ancona, Italy

**Keywords:** Neutrophil, Lymphocyte, Ratio, Prognosis, Survival, Triple negative, Breast cancer

## Abstract

**Background:**

There is a growing body of evidence that immune response plays a large role in cancer outcome. The neutrophil to lymphocyte ratio (NLR) has been used as a simple parameter of systemic inflammation in several tumors. The purpose was to investigate the association between pre-treatment NLR, disease-free survival and overall survival in patients with early triple negative breast cancer (TNBC).

**Methods:**

We reviewed the records of patients with stage I-III TNBC at our Institution from 2006 to 2012. The association between pre-treatment NLR and survival was analyzed. The difference among variables was calculated by chi-square test. DFS and OS were estimated using Kaplan-Meier method. Cox analysis was performed to analyze clinical parameters for their prognostic relevance.

**Results:**

A total of 90 patients were eligible. There was no significant correlation among pre-treatment NLR and various clinical pathological factors. Patients with NLR higher than 3 showed significantly lower DFS (p = 0.002) and OS (p = 0.009) than patients with NLR equal or lower than 3. The Cox proportional multivariate hazard model revealed that higher pre-treatment NLR was independently correlated with poor DFS and OS, with hazard ratio 5.15 (95% confidence interval [CI] 1.11-23.88, p = 0.03) and 6.16 (95% CI 1.54-24.66, p = 0.01) respectively.

**Conclusion:**

Our study suggests that pre-treatment NLR may be associated with DFS and OS patients with early TNBC. Further validation and a feasibility study are required before it can be considered for clinical use.

## Background

Triple negative breast cancer (TNBC) represents approximately 10–20% of breast cancers and they are defined by the absence of estrogen receptor, progesterone receptor and human epidermal growth factor receptor-2 expression. Recurrence and disease progression are relatively common for women with TNBC, with a peak risk of recurrence within the first three-five years after diagnosis. A large tumour size, nodal involvement and poor clinical outcomes for women with TNBC may in part be explained by intrinsically aggressive tumour pathology, including high mitotic index, high histologic grade, high proliferation, and a high frequency of TP53 mutations associated with a frequent occurrence of visceral metastases and poor prognosis [1,2].

Owing to the aggressive tumor biology and lack of targeted therapy, TNBC is characterized by a dismal although heterogeneous outcome. Recently, considerable efforts have been made to sub-classify TNBC into different prognostic groups. In 2011, Lehman et al analysed gene expression (GE) profiles identifying 587 TNBC cases. Particularly, cluster analysis identified 6 TNBC subtypes displaying unique GE and ontologies, including 2 basal-like (BL1 and BL2), an immunomodulatory (IM), a mesenchymal (M), a mesenchymal stem–like (MSL), and a luminal androgen receptor (LAR) subtype [3]. These data will be necessary in biomarker selection and drug discovery, but in clinical practice GE analysis is not available to define TNBC with more aggressive behaviour and poor prognosis [3-6]. Nevertheless, new laboratory factors should be accurate and reproducible, but also easily performed. Increasing evidence supports the involvement of inflammation in cancer development, progression, metastasis and relapse [7,8]. The combined index, using neutrophil and lymphocyte counts in the form of neutrophil to lymphocyte ratio (NLR), has been used as simple parameter to assess the systemic inflammation. It is correlated with prognosis in several tumors, such as colorectal, gastric, pancreatic, non-small-cell lung, hepatocellular, ovarian, cervical and renal cancers [9-16]. Previous studies have investigated the role of NLR in predicting survival and mortality even in early breast cancer patients [17-19]. Based on the lack of any clinical prognostic features predicting prognosis in the subgroup of TNBC, the purpose of this study was to investigate the association between pre-treatment NLR, disease-free survival (DFS) and overall survival (OS) in patients with early TNBC.

## Methods

### Patients

We retrospectively identified patients who were diagnosed and completed the treatment of invasive breast cancer at our institution from January 2006 to December 2012. The study obtained the necessary approval by the Department of Medical Oncology, AO Ospedali Riuniti, Ancona. According to our country’s legislation, since it was a retrospective study, with no direct patient involvement, the ethical approval and patients consent for the study were not required (Official Gazette No. 72 of March 26, 2012). Medical record were reviewed to find data on patient’s medical history, age, sex, pathologic results such as tumour size, lymph node status, hormonal status, human epidermal growth factor receptor 2 (HER-2), receptor status and laboratory data. Patients with ductal carcinoma *in situ* with or without micro-invasion and patients with lack of information on pathologic or laboratory results were excluded. We also excluded patients with stage IV breast cancer or inflammatory breast cancer, patients who were diagnosed preoperatively with systemic inflammatory or chronic disease such as systemic lupus erythematosus (SLE), any haematological disorders, liver cirrhosis, end-stage renal disease, pregnancy-related breast cancer, treatment with statins, steroids or cytokines or granulocyte stimulating factor (G-CSF).

Patients were eligible if they had Eastern Cooperative Oncology Group performance status 0-2; age between 18 and 80; no history of diabetes, heart failure, coronary artery disease, hypertension, cerebrovascular disease and peripheral arterial diseases; adequate bone marrow and organ functions (WBC >4.000/mm^3^ and or absolute neutrophil count (ANC) >1.500/mm^3^; platelets >100.000/mm^3^; AST/ALT <2.5 times the upper normal limit (UNL); bilirubin <2 mg/dl; creatinine <1.5 mg/dl).

### Pathological characteristics

Based on pathology reports, we identified tumors lacking immunohistochemical expression of oestrogen receptor (ER), progesteron receptor (PR) and HER2. ER and PR were considered positive if there were at least 1% positive invasive tumor nuclei in the sample. HER-2 status was evaluated by immunohistochemistry (IHC) using a semiquantitative score (0–3+). Tumor staining was compared to the staining of normal breast epithelium from the same patient as a negative control. For clinical purposes, no staining or weak (1+) and incomplete membrans’ staining was considered a negative result. Patients with 2+ IHC staining for HER2 underwent fluorescence *in-situ* hybridization to confirm HER2 negativity. Triple-negative status (ER negative, PR negative and HER-2 negative) was finally diagnosed and re-reviewed by the single study pathologist of our Institution. Rare histological types of TNBC (apocrine, medullary, adenoid cystic and metaplastic carcinomas) were excluded from this analysis.

### Laboratory data

**Figure 1:**
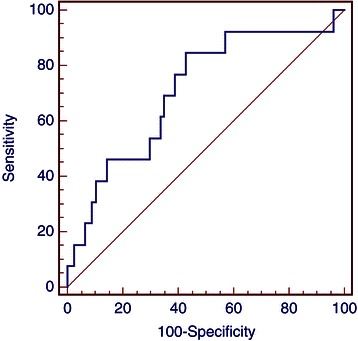
**Receiver operating characteristics (ROC) analysis based on NLR for DFS.** In this model sensitivity was 84.6% (95% CI 54.5 – 97.6) and specificity was 57.1% (95% CI 45.4–68.4). AUC was 0.71, p = 0.01.

**Figure 2:**
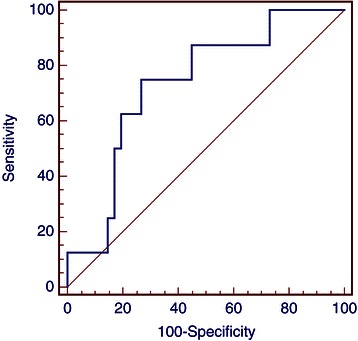
**Receiver operating characteristics (ROC) analysis based on NLR for OS.** In this model sensitivity was 75% (95% CI 35.0 – 96.1) and specificity was 73.1% (95% CI 62.2–82.4). AUC was 0.73, p = 0.02.

The NLR was defined as the absolute neutrophil count divided by absolute lymphocyte count. The NLR was calculated from the full blood count routinely performed immediately after breast cancer diagnosis and before the initiation of any treatment modality, including surgery (pre-treatment NLR). The cut-off value of 3 was decided as the maximum (sensitivity + specificity) point according to receiver operating characteristics curves (Figures 1 and 2). Patients were further divided into two groups, A (NLR ≤ 3) and B (NLR > 3).

### Statistical analysis

Patients who were not reported as died at the time of the analysis were censored at the date they were last known to be alive. Disease-free survival (DFS) was defined as the interval between the date of diagnosis of TNBC to the first failure (including locoregional and/or distant relapse, second primary or death). Overall survival (OS) was defined as the interval between histological diagnosis to death or last follow-up visit. Survival distribution was estimated by the Kaplan—Meyer method. The association between categorical variables was estimated by Chi square test. The Cox multivariate proportional hazard regression model was used to evaluate the effects of the prognostic factors on survival. Significant differences in probability of surviving between the strata were evaluated by log-rank test. Hazard ratios and 95% confidence intervals (CIs) were estimated from regression coefficients. A significance level of 0.05 was chosen to assess the statistical significance. Statistical analysis was performed with MedCalc package (MedCalc® v9.4.2.0).

## Results

**Figure 3:**
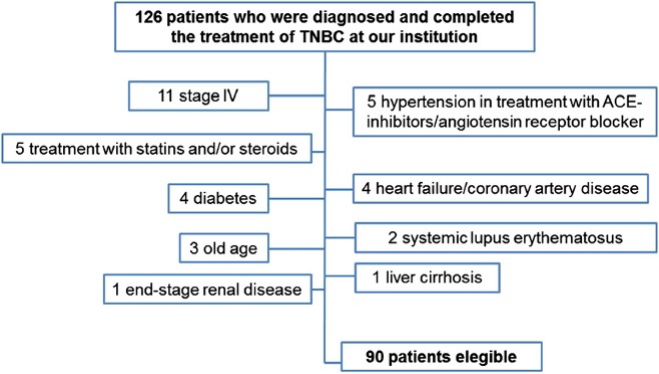
**We identified 126 patients who were diagnosed and completed the treatment of triple-negative breast cancer; 90 patients were eligible for analysis.** ACE = Angiotensin-Converting Enzyme inhibitors.

**Figure 4:**
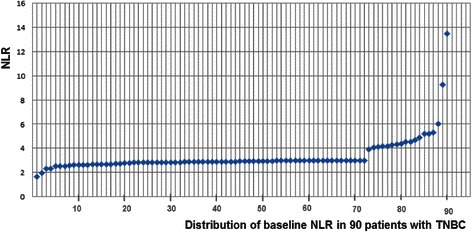
**Distribution of the baseline NLR in the peripheral blood of 90 patients with triple negative breast cancer (TNBC).**

We identified 126 patients who were diagnosed and completed the treatment of TNBC; a total of 90 patients were eligible for analysis. The reasons for the excluded patients are summarized in Figure 3. The median value of NLR was 2.93 (range 1.62-13.47). The distribution of the baseline NLR of the 90 patients is shown in Figure 4. 17 patients (18.9%) showed higher pre-treatment NLR (group B). Median age at diagnosis was 53 years (range 28-79). The median follow-up time was 53.8 months (13.1-95.2). Pathological T stage was T1 in 52 and T2-T3 in 38 patients. Lymph nodes were disease-positive in 42.3% of cases. Ductal tumors (91.1%), a grading of 3 (90%) and a high proliferative index (Ki-67 > 20%) (83.4%) were the most commonly observed categories. Vascular invasion and necrosis were found in 15.6% and 16.6% of patients, respectively.

**Figure 5:**
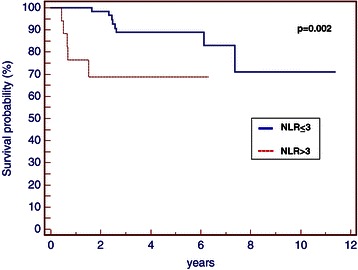
**DFS of patients with early TNBC based on NLR (p = 0.002).**

**Figure 6:**
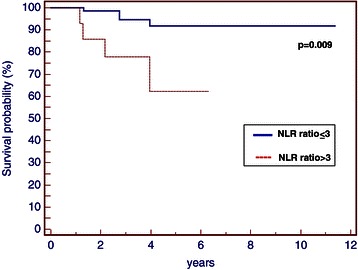
**OS of patients with early TNBC based on NLR (p = 0.009).**

There was no significant correlation among pre-treatment NLR and various clinical pathological factors, including age, menopausal status, tumour size, lymph nodes status, grading, Ki-67, necrosis and lympho-vascular invasion (Table 1). Patients with NLR equal to or higher than 3 showed significantly lower 5-year disease-specific survival rate than patients with NLR lower than 3 (5-year survival, 88.8% vs. 68.8%; p = 0.002) (Figure 5). The patients with NLR equal to or higher than 3 were associated with increased breast cancer specific mortality (5-year overall survival, 91.9% vs. 62.3%; p = 0.009) (Figure 6), than patients with NLR equal or lower than 3. A better OS was also correlated to the absence of necrosis (p = 0.003). The Cox proportional multivariate hazard model revealed that higher pre-treatment NLR was independently correlated with poor DFS and OS, with hazard ratio 5.15 (95% confidence interval [CI] 1.11-23.88, p = 0.03) and 6.16 (95% CI 1.54-24.66, p = 0.01) respectively. Multivariate statistical analysis also confirmed necrosis as an independent prognostic variable influencing OS (p = 0.01; HR = 6.92, 95% 1.48-32.35) (Tables 2 and 3).

**Table 1:** **Baseline characteristics of 90 patients with TNBC by NLR**

Characteristics	Total (n = 90)	NLR ≤3 (n = 73)	NLR > 3 (n = 17)	p-value
Age				
≤50 years	41 (45.5)	35 (38.8)	6 (6.7)	0.17
>50 years	49 (54.5)	38 (42.3)	11 (12.2)	
Performance status				
ECOG 0	70 (77.7)	62 (68,8)	8 (8.9)	0.37
ECOG 1	20 (22.3)	11 (12.3)	9 (10.0)	
Menopausal status				
Pre-	36 (40.0)	31 (34.4)	5 (5.6)	0.44
Post-	54 (60.0)	42 (46.7)	12 (13.3)	
Tumour size				
pT1	52 (57.7)	45 (49.9)	7 (7.8)	0.56
pT2	37 (41.1)	27 (30.0)	10 (11.1)	
pT3	1 (1.2)	1 (1.2)	0 (0)	
Lymph node status (pN)				
pN0	52 (57.7)	42 (46.6)	10 (11.1)	0.44
pN1	28 (31.2)	24 (26.7)	4 (4.5)	
pN2	10 (11.1)	7 (7.8)	3 (3,3)	
Stage^*^				
I	33 (36.7)	29 (32.2)	4 (4.5)	0.39
II	48 (53.3)	38 (42.2)	10 (11.1)	
IIIA	9 (10.0)	6 (6.7)	3 (3.3)	
Tumour histology				
Ductal carcinoma	82 (91.1)	69 (76.7)	13 (14.4)	0.29
Lobular carcinoma	1 (1.2)	0 (0)	1 (1.2)	
Other	7 (7.7)	4 (4.4)	3 (3.3)	
Histologic grade				
I-II	9 (10.0)	7 (7.8)	2 (2.2)	0.84
III	81 (90.0)	66 (73.3)	15 (16.7)	
Ki-67				
≤20%	15 (16.6)	13 (14.4)	2 (2.2)	0.79
>20%	75 (83.4)	60 (66.7)	15 (16.7)	
Lympho-vascular invasion				
Yes	14 (15.6)	7 (7.8)	7 (7.8)	0.07
No	76 (84.4)	66 (73.3)	10 (11.1)	
Necrosis				
Yes	15 (16.6)	11 (12.1)	4 (4.5)	0.89
No	75 (83.4)	62 (69.0)	13 (14.4)	
Type of surgery				
Quadrantectomy	71 (77.9)	60 (65.7)	11 (12.2)	0.75
Radical mastectomy	19 (22.1)	13 (15.4)	6 (6.7)	
Adjuvant chemotherapy				
Antracycline containing	48 (53.3)	40 (44.4)	8 (8.9)	0.59
CMF	40 (44.5)	31 (34.5)	9 (10.0)	
No	2 (2.2)	2 (2.2)	0 (0)	
Adjuvant radiotherapy				
Yes	71 (77.9)	60 (65.7)	11 (12.2)	0.61
No	19 (22.1)	13 (15.4)	6 (6.7)	
Recurrences				
Yes	13 (14.5)	8 (8.9)	5 (5.6)	0.12
No	77 (85.5)	65 (72.2)	12 (13.3)	
Deaths				
Yes	8 (8.9)	4 (4.4)	4 (4.5)	0.06
No	82 (91.1)	69 (76.7)	13 (14.4)	

Table 1 shows the lack of significant correlation among pre-treatment NLR and clinical pathological factors.

*AJCC. Cancer Staging manual. Seventh edition. New York, Springer 2009.

Legend: NLR = Neutrophil to Lymphocyte Ratio; TNBC = Triple Negative Breast Cancer.

**Table 2:** **Cox regression analysis for disease-free survival in TNBC**

Parameters	Univariate	Multivariate	
	p-value	HR, 95% CI	p-value
Age (≤50 years vs >50 years)	0.12	2.18 (0.02-143.2)	0.74
Menopausal Status (Pre- vs Post-)	0.15	0.80 (0.01-51.3)	0.91
Tumour size (pT1 vs pT2-T3)	0.08	1.81 (0.45-7.14)	0.39
Lymph node status (pN0 vs pN+)	0.2	1.25 (0.31-4.96)	0.74
Nuclear Grade (G1-G2 vs G3)	0.08	0.66 (0.27-1.62)	0.37
Ki-67 (≤20% vs >20%)	0.38	0.78 (0.27-2.26)	0.66
Lympho-vascular invasion (absence vs presence)	0.17	1.68 (0.26-10.70)	0.58
Necrosis (absence vs presence)	0.08	3.75 (0.69-20.15)	0.12
Intraductal carcinoma (absence vs presence)	0.9	1.87 (0.43-8.10)	0.40
NLR (≤3 vs >3)	**0.002**	5.15 (1.11 – 23.88)	**0.03**

Table 2 shows a significant correlation between DFS and higher pre-treatment NLR.

Legend: NLR = Neutrophil to Lymphocyte Ratio; HR = hazard ratio; CI = confidence interval; TNBC = Triple Negative Breast Cancer.

**Table 3:** **Cox regression analysis for overall survival in TNBC**

Parameters	Univariate	Multivariate	
	p-value	HR, 95% CI	p-value
Age (≤50 years vs >50 years)	0.06	1.79 (0.04-136.2)	0.68
Menopausal Status (Pre- vs Post-)	0.08	1.18 (0.08-127.6)	0.93
Tumour size (pT1 vs pT2-T3)	0.06	2.10 (0.30-14.4)	0.44
Lymph node status (pN0 vs pN+)	0.05	4.29 (0.65-28.13)	0.13
Nuclear Grade (G1-G2 vs G3)	0.28	0.64 (0.10-3.84)	0.62
Ki-67 (≤20% vs >20%)	0.75	1.49 (0.21-10.30)	0.68
Lympho-vascular invasion (absence vs presence)	0.16	4.95 (0.60-40.30)	0.13
Necrosis (absence vs presence)	**0.009**	6.92 (1.48-32.35)	**0.01**
Intraductal carcinoma (absence vs presence)	0.94	1.36 (0.22-8.17)	0.73
NLR (≤3 vs >3)	**0.003**	6.16 (1.54 – 24.66)	**0.01**

Table 3 shows a significant correlation between OS and either higher pre-treatment NLR and necrosis.

Legend: NLR = Neutrophil to Lymphocyte Ratio; HR = hazard ratio; CI = confidence interval.

## Discussion

Inflammation is involved in breast cancer development, tumor angiogenesis and progression. The pro-tumorigenic activity mediated by immune system cells and associated inflammatory mediators, is countered by antitumor immunity [20,21]. Moreover, recent studies suggested that inflammation could be also responsible for treatment resistance during therapy [22,23] and even involved in relapse and metastasis process in breast cancer, promoting the angiogenic switch [24-27]. Furthermore, it has been shown that the presence of a lymphocytic infiltrate in several tumor types could be considered a predictor of a favourable outcome. In breast cancer tumor-infiltrating lymphocytes is associated with a better survival, a better response to anthracycline-based chemotherapy, as well as better response to neoadjuvant chemotherapy [28,29]. Recent studies have identified different immune response signature, based on the combination of high levels of tumor-associated macrophages, robust Th2 responses, and low CTL/NK cell infiltration, in breast cancer correspondent to the molecular profiles, that could provide useful information on patient prognosis [30,31]. Several studies have also investigated the relation between systemic inflammation and breast cancer survival, reporting a significant association between shorter survival and elevated concentration of circulating inflammatory biomarkers, such as serum amyloid A (SSA) and systemic c-reactive protein (CRP) and serum interleukin-6 [32,33].

NLR is a routinely available marker of the systemic inflammatory response. The derived NLR (dNLR) and NLR have recently been shown to negatively influence the clinical outcome in various cancer entities.

NLR has been previously evaluated in different settings of patients with breast cancer. In a large cohort of 442 patients observed that only in luminal A patients NLR (>2.5) was able to identify a poor prognosis [17]. Similar results were reported by Azab et al in 316 BC patients. In the highest NLR quartile (NLR >3.3) showed a significant increase in all-cause mortality rate at 1-,2- and 5-year follow-up compared with the lowest three NLR quartiles, suggesting that NLR is an independent, significant predictor of short- and long-term mortality in BC patients [18]. In a recent retrospective analysis, NLR continued to be statistically significant predictor of 5-year mortality in all lymphocyte count subsets, even better than PLR (platelet to lymphocyte ratio) [19].

We investigated the prognostic role of pre-treatment NLR in TNBC subtype and our study suggests that increased pre-treatment NLR may be associated with worse DFS and OS in patients with early TNBC. The role of the neutrophils/lymphocyte ratio could represent a new accurate and reproducible laboratory index to identify TNBC patients with poorer prognosis.

Further, our data are consistent with several previous studies conducted in a variety of solid organ malignancies including gastro-intestinal cancers, gynaecological cancers, non-small cell lung cancer, urological cancers and soft-tissue sarcoma, in which NLR has been reported to have a prognostic value [11]. In particular, there is increasing evidence supporting the associations between pre-operative NLR and outcome in patients with operable disease, in particular gastrointestinal cancer, pancreatic cancer and in hepatocellular carcinoma [9-11].

The NLR was consistently associated with overall and disease-free survival in several studies in this setting of patients with operable disease on univariate analysis, although the role as independent prognostic factor was not always confirmed. A number of studies failed to report a relation between NLR and clinical-pathological features, such as tumour size, microvascular and lymphatic invasion, lymph node involvement, number of metastatic lesions and elevated bio-marker concentration [11]. Interestingly, Wang and colleagues reported that the NLR was significantly associated with markers of functional decline, including poor performance status and weight loss, in patient with pancreatic cancer [34].

Otherwise, literature data agree that NLR reliably predicts poorer survival in more advanced states such as those patients requiring chemotherapy or who have inoperable disease and, together with other systemic inflammation-based scores, is a surrogate index of progressive nutritional and functional decline in the cancer patients. [35-38]. The threshold most used to define an elevated NLR was >5, but several analysis used also threshold between 2.5 and 4.

Previous studies have investigated the role of NLR in predicting survival and mortality in early breast cancer patients. Noh and colleagues showed that patients with NLR equal to or higher than 2.5 showed significantly lower 5-year and 10-year disease-specific survival rate than patients with NLR lower than 2.5. Further, patients with higher NLR equal to or higher than 2.5 were associated with increased T stage, younger age, positive HER2 status, and higher disease-specific mortality [17]. On the other hand, Azab and colleagues divided patients enrolled in their analysis in four quartiles; the highest NLR quartile (NLR > 3.3) had higher 1-year and 5-year mortality rates compared with those in the lowest quartile (NLR < 1.8) [18].

Circulating granulocyte neutrophil cells count, at the numerator, were been shown to contain and secrete the majority of cytokines, such as vascular endothelial growth factor (VEGF), interleukin-18 (IL-18) and matrix metalloproteinases (MMM), that create the optimal environment for tumor growth, progression and metastasis [39-41]. Neutrophilia is already considered as adverse outcome predictor in several tumors [18,42,43]. On the other hand, cytotoxic T Lymphocytes (CTL) are known to induce apoptosis of cancer cells and inhibit tumor growth while CD8+ T lymphocyte infiltration is associated with better overall patient outcomes. However, the lymphocyte count and the neutrophil absolute count, that represent the denominator and the numerator respectively, are greatly influenced by various physiological, pathological and physical factors; NLR superiority is due to the stability of the ratio compared with the absolute cellular counts [18].

Furthermore, our data showed a correlation between OS and necrosis in the histological sample; in particular the absence of necrosis was associated with a better outcome in our patients and necrosis was an independent prognostic variable influencing OS. Actually, necrosis is usually considered to be immunologically harmful because of the sudden release of proinflammatory mediators. Necrotic cell death causes the release of proinflammatory cytokines, such as IL-8, IL-10, TNF-alpha or of terminal mediators of inflammation, known to promote recruitment of inflammatory cells and to induce the cytokines and chemokines cascade. Therefore, necrosis could represent a link between inflammation and stromagenesis, angiogenesis, and suppression of the adaptive immune response, mechanisms involved in tumor growth, and could be charge also in cell resistance to therapy [24].

In particular, several studies suggest that NF-jB activation by a proinflammatory tumor microenvironment can promote an aggressive breast cancer phenotype through activating or suppressing ERa target gene expression [44] and recently it was showed to be involved in endocrine therapy resistance [45]. Furthermore, epidemiologic studies showed that regular use of nonsteroidal anti-inflammatory drugs could reduce the risk of ERa + breast cancer; it was not demonstrated for ERa - breast cancers [46].

We are aware of some limitations in our study. It is a retrospective analysis in a single institution, on a small number of patients. Further several other conditions that can be potentially affect the measurement of NLR were not taken into account in our analysis, such as metabolic syndrome, abnormal thyroid function tests, smoking, alcohol consumption and hypercolesterolemia [47].

However, to our knowledge, it is the first analysis showing that pre-treatment NLR could predict DFS and OS in TNBC patients. Because of the lack of any other clinical prognostic features, further validation work and feasibility study are required before the results of this study can be considered for clinical use. Finally, neutrophil to lymphocyte ratio is an inexpensive, easy to obtain, widely available marker of inflammation and could be integrated with other factors, such as the platelet:lymphocyte ratio, to derive simple inflammation-based prognostic scores, such as the Glasgow Prognostic [48].

Other interesting evidence are emerging about the role of the tumor- infiltration immunophenotype in TNBC in predict clinical outcome [28,49-51], which should be interestingly integrated with our data. Prospective studies are needed to determine the immunogenic mechanisms underlying NLR variations and to adequately assess the potential role of NLR in guiding patient selection and treatment decisions. Groups defining staging for neoplasms are strongly encouraged to assess and incorporate measures of the presence of apoptosis, autophagy, and necrosis as well as the nature and quality of the immune infiltrate.

## Conclusion

Our study suggests that pre-treatment NLR may be associated with DFS and OS patients with early TNBC and can be easily introduced in clinical practice in order to identify TNBC patients with poor prognosis. Prospective studies are needed to assess the potential role of NLR in guiding treatment decisions, patient selection and clinical trial design.

However, it needs to be validated in larger prospective studies for it to be useful in risk stratification.

## Abbreviations

CTL: Cytotoxic T Lymphocytes
CRP: Systemic C-reactive protein
DFS: Disease free-survival
ER: Oestrogen receptor
HER-2: Human epidermal growth factor receptor 2
IHC: Immunohistochemistry
IL-18: interleukin-18
MMM: Matrix metalloproteinases
NLR: Neutrophil to lymphocyte ratio
dNLR: Derived neutrophil to lymphocyte ratio
OS: Overall survival
PLR: Platelet to lymphocyte ratio
PR: Progesteron receptor
SLE: Systemic lupus Erythematosus
SSA: Serum amyloid A
TNBC: Triple negative breast cancer
VEGF: Vascular endothelial growth factor
